# Home-quarantine during the initial Covid-19 outbreak in Israel: parent perceived impact on children with ASD

**DOI:** 10.1016/j.heliyon.2022.e09681

**Published:** 2022-06-08

**Authors:** Ayelet Arazi, Judah Koller, Ditza A. Zachor, Ofer Golan, Yair Sadaka, Dganit Eytan, Orit Stolar, Naama Atzaba-Poria, Hava Golan, Idan Menashe, Gal Meiri, Lidia V. Gabis, Ilan Dinstein

**Affiliations:** a Department of Cognitive & Brain Sciences, Ben Gurion University of the Negev, Beer Sheva, Israel; b Azrieli National Centre for Autism and Neurodevelopment Research, Ben Gurion University of the Negev, Beer Sheva, Israel; c Seymour Fox School of Education, The Hebrew University of Jerusalem, Jerusalem, Israel; d Sackler Faculty of Medicine, Tel Aviv University, Tel Aviv, Israel; e The Autism Center/ALUT, Shamir (Assaf Harofeh) Medical Center, Israel; f Autism Treatment and Research Center – Association for Children at Risk, Ramat Gan, Israel; g Department of Psychology, Bar-Ilan University, Ramat Gan, Israel; h Autism Research Centre, Department of Psychiatry, University of Cambridge, UK; i Neuro-Developmental Research Centre, Beer Sheva Mental Health Centre, Ministry of Health, Beer Sheva, Israel; j ALUT – The Israeli Society for Children and Adults with Autism, Ramat Gan, Israel; k School of Education, Bar-Ilan University, Ramat Gan, Israel; l Department of Psychology, Ben Gurion University of the Negev, Beer Sheva, Israel; m Department of Physiology and Cell Biology, Faculty of Health Sciences, Ben Gurion University of the Negev, Beer Sheva, Israel; n Department of Public Health, Ben Gurion University of the Negev, Beer Sheva, Israel; o Pre-School Psychiatry Unit, Soroka University Medical Center, Beer Sheva, Israel; p Sagol School of Neuroscience, Tel Aviv University, Tel Aviv, Israel; q Child Development Services, Maccabi Healthcare, Tel Aviv, Israel; r Duet Center, Ben Gurion University of the Negev, Beer-Sheva, Israel

**Keywords:** Covid-19, Quarantine, Lockdown, Corona, Psychological impact, Support

## Abstract

**Background:**

Studies have reported that Covid-19 home-quarantine periods have had mostly negative psychological impact on children with ASD and their families. Here we examined parent perceived impact of a 6-week quarantine period imposed in Israel at the beginning of the Covid-19 outbreak, in mid-March 2020.

**Methods:**

An anonymous online questionnaire was completed by parents of 268 children with ASD. Parents rated deterioration/improvement in their child's behaviors, abilities, mood, sleep, and anxiety along with changes in their own mood, sleep, parenting skills, and family relationships. We performed t-tests and ANOVA analyses to assess the significance of perceived impact on each domain and potential differences in the impact across families with children of different ages, genders, and levels of required support as well as families that experienced different magnitudes of economic hardships.

**Results:**

Parents reported significant deterioration in their mood and sleep along with significant improvements in relationships with their spouse and child with ASD, and in their parenting skills. Parents also reported significant increases in the severity of tantrums, anxiety, and restricted and repetitive behavior symptoms along with significant improvements in social and communication abilities of their child with ASD. Ratings were significantly lower in families of ASD children who regularly require more support and in families that experienced economic hardships.

**Conclusions:**

While periods of home-quarantine create numerous hardships for families of children with ASD, they may also offer an opportunity for improving parenting skills, family relationships, and children's social communication abilities with potential relevance for improving remote services.

## 1 Introduction

In mid-March 2020 approximately 100 new cases of Covid-19 were diagnosed daily in Israel [1] and the World Health Organization declared COVID-19 as a pandemic [2]. This motivated the Israeli government to implement a nation-wide mandatory home-quarantine period that lasted until early-May 2020 [3]. All non-essential businesses and services were closed along with the national education system including all special education services [4]. The general population, excluding individuals with essential jobs, was ordered to stay within 100m of their home except for brief excursions for receiving medical services, replenishing food supplies, and other rare circumstances. In this situation, parents throughout the country were the sole caregivers for their children and support services were partially available only through online or social media platforms.

The closure had immediate economic ramifications, as reported by the Israeli Central Bureau of Statistics, with >20% of the Israeli work force losing their jobs or being put on “temporary leave” [5]. While the Israeli government implemented several economic aid packages, many Israeli families experienced a considerable reduction in income.

Previous studies of the general population have reported that quarantine periods cause a variety of negative psychological effects [6] including increased economic related anxiety [7], general anxiety [8], depression [9], and post-traumatic stress disorder (PTSD) symptoms [10]. Initial reports demonstrate that Covid-19 lockdowns have indeed caused an increase in anxiety, depression, PTSD, and insomnia symptoms in the general population [11]. This includes increased economic anxiety [12, 13] particularly in parents with young children [14]. It has been suggested that the negative psychological impact of quarantine periods is likely to be larger for families of children with ASD who usually rely on multiple support services to manage daily activities and deal with behavioral and clinical challenges associated with ASD [15, 16, 17].

Despite the many difficulties and negative psychological outcomes associated with home-quarantine, there may also be positive consequences to isolating families at home. Spending time with parents is thought to be one of the most important pillars of child development, enabling the establishment of secure attachment and the development of healthy social and cognitive abilities [18, 19]. Indeed, a variety of studies have suggested that decreasing parental time in modern society, due to ever-growing occupational demands, has negative impact on child development [20, 21]. During home-quarantine periods parents have the opportunity to spend more time engaging their children in educational, social, and play activities, which are particularly important for development [20, 21]. Hence, home-quarantine may offer a unique opportunity for improving parent-child relationships, parenting skills, and consequent child development.

Recent studies that have specifically assessed the impact of Covid-19 lockdowns on individuals with ASD, have mostly focused on their negative impact. This includes reports of increased anxiety and depression in adults with ASD [22], in children and adolescents with ASD, and in their parents [23, 24]. In addition, parents reported that children with ASD exhibited increased aberrant behaviors [25], sleep difficulties, and lethargy [26] following periods of home-quarantine.

Nevertheless, without detracting from the clear challenges noted above, several studies have reported that Covid-19 lockdowns also had positive impact on children with ASD and their families. Specifically, parents in Spain reported that they were able to spend more time with their children, improve their parenting skills, and witnessed improvements in the participation and communication abilities of their children with ASD [27]. Some preschool children with ASD even exhibited improvements in formal assessments of adaptive behaviors following the initial Covid-19 home-quarantine period in Italy [28]. These studies demonstrate that home-quarantine may also have positive impact on children with ASD and their parents.

The purpose of this exploratory study was to quantify parental perceptions of the impact of the initial 6-week quarantine period in Israel. We hypothesized that this unprecedented quarantine period may have positive or negative impact on specific core ASD symptoms as well as the child's behavior, mood, anxiety, and sleep. In addition, we hypothesized that the quarantine would impact parent relationships with spouse and child as well as the parents' mood, sleep, and parenting skills. Finally, we examined whether the quarantine impact differed across families with children of different ages, genders, and levels of required support as well as across families that experienced different magnitudes of economic hardships during the quarantine.

## 2 Methods

### 2.1 Procedure and participants

The questionnaire was available online at the end of the home-quarantine period between 5-20^th^ of May 2020, as restrictions were slowly being lifted and the education systems were gradually re-opening in Israel. The questionnaire was advertised by the Azrieli National Centre for Autism and Neurodevelopment Research on Israeli social media channels frequented by parents of children with ASD. Parents of 268 children with ASD completed the questionnaire. The mean age of participating children was 7.6 ± 4.3 years old (range 2–18) with 215 males (80%). The mean age of the parents who completed the questionnaire was 41 ± 6.4 years old (range 26–61). All participants acknowledged a consent statement allowing use of their responses for research, questionnaires were completed anonymously, and participants were not compensated. Ethical approval for the study was granted by the Ben Gurion University Human Subjects Research Committee.

### 2.2 Questionnaire

The questionnaire was constructed during the initial Covid-19 quarantine period by the authors to assess parent perception of changes in their child's core and secondary ASD symptoms as well as general functioning. In addition, parents reported on their own mood and sleep, as well as their parenting abilities and relationships with their child and partner. Questions were novel and created by the authors based on our extensive research experience with validated questionnaires such as the social responsiveness scale [29], adaptive behavior analysis system [30], aberrant behaviors checklist [31], child sleep habits questionnaire [32], parent stress index [33], couples' satisfaction index [34], and others. Given the need to quickly deploy the questionnaire we did not have time to validate it against these existing questionnaires or to assess inter-rater reliability.

To report the positive or negative impact of the quarantine period, parents used an 11-point scale ranging from very negative impact to very positive impact. The questionnaire consisted of four parts (see full version in Supplementary Materials):

1. General information – This section included 14 questions about the age and gender of child and parent, the amount of daily support that the child requires (low, medium, or high), and the intensity of intervention, quantified by the total number of intervention hours that the child received before the quarantine (e.g., speech therapy, physical therapy, etc..).
2. Impact on the parent – Parents reported the financial impact of the quarantine as: severe (>40% income reduction), moderate (20–40% reduction), minor (<20% reduction), or none (no reduction). Parents then rated the quarantine impact on the atmosphere at home, relationship with domestic partner, parenting co-operation, parenting skills, relationship with child with ASD, enjoyment with child with ASD, enjoyment with other children, quality and quantity of sleep, and general mood.
3. Impact on the child with ASD – Parents rated the quarantine impact on their child's general mood, ability to perform daily activities (e.g., dressing, brushing teeth), eating and nutrition, quality and quantity of sleep, fear/anxiety, frequency and magnitude of tantrums, sensory problems, verbal communication, non-verbal communication, reciprocal play, quantity and severity of repetitive/stereotypical behaviors, and difficulties with changes in daily routines.
4. Online services and interventions – Parents rated the amount and helpfulness of online services that the family received during the quarantine period.

### 2.3 Grouping individual questions into domains

Individual questions/items were grouped according to conceptual and theoretical criteria into four child related domains and three parent/family related domains that are commonly studied in the autism and child development fields. Two child related domains focused on each of the core ASD symptoms as defined by the DSM-5 [35]: social communication skills and restricted and repetitive behaviors. Two additional domains included the child's general functioning and aberrant behaviors, which are often described as secondary ASD symptoms. Questions included in each of the four child domains exhibited high internal reliability as measured by Cronbach's alpha:

Child's general functioning included four ratings: the child's general mood, independence in performing daily activities, feeding and nutrition, and amount and quality of sleep. Cronbach's alpha = 0.84.

Child's aberrant behaviors included three ratings: the amount and severity of outbreaks/tantrums, fears and anxiety, and sensory problems. Cronbach's alpha = 0.87.

Child's restricted and repetitive behaviors (RRB) included two ratings: difficulties with changes in daily routines and the amount and severity of repetitive/stereotypical behaviors. Cronbach's alpha = 0.75.

Child's social communication skills included three ratings: reciprocal play, verbal communication, and non-verbal communication. Cronbach's alpha = 0.9.

Parent related domains focused on the relationship with the partner and the general atmosphere at home, parenting skills and relationships with children, and the parent's mood and sleep. Questions included in each of the three parent domains also exhibited high internal reliability as measured by Cronbach's alpha:

Atmosphere at home included three ratings: atmosphere at home, relationship with domestic partner, and cooperation between partners. Cronbach's alpha = 0.79.

Parent skills and relationship with children included four ratings: parental skills, relationship with child with ASD, enjoyment from interactions with child with ASD, enjoyment from interactions with other children at home. Cronbach's alpha = 0.88.

Parent mood and sleep included two ratings: general mood of the parent and the amount and quality of their sleep. Cronbach's alpha = 0.79.

The rating of each domain was computed as the mean of the ratings composing it.

### 2.4 Statistical analyses

Scores from each of the items or domains were analyzed using one sample, two tailed t-tests. In the 11 point rating scale, a score of zero indicated no change while scores from +1 to +5 indicated increasing magnitudes of positive impact (i.e., improvement) and scores from -1 to -5 indicated increasing magnitudes of negative impact (i.e. deterioration). A separate t-test was applied to assess the significance of change on each item and we corrected for multiple comparisons using Bonferroni, separately for the 9 parent-related and 12 child-related items. We used one-way ANOVA analyses to test for significant differences across families that differed with respect to the child's age, amount of required support, economic impact, or frequency/efficiency of online consultations. These analyses were followed by post-hoc Tukey's test when the ANOVA results indicted significant differences.

## 3 Results

**Figure 1:**
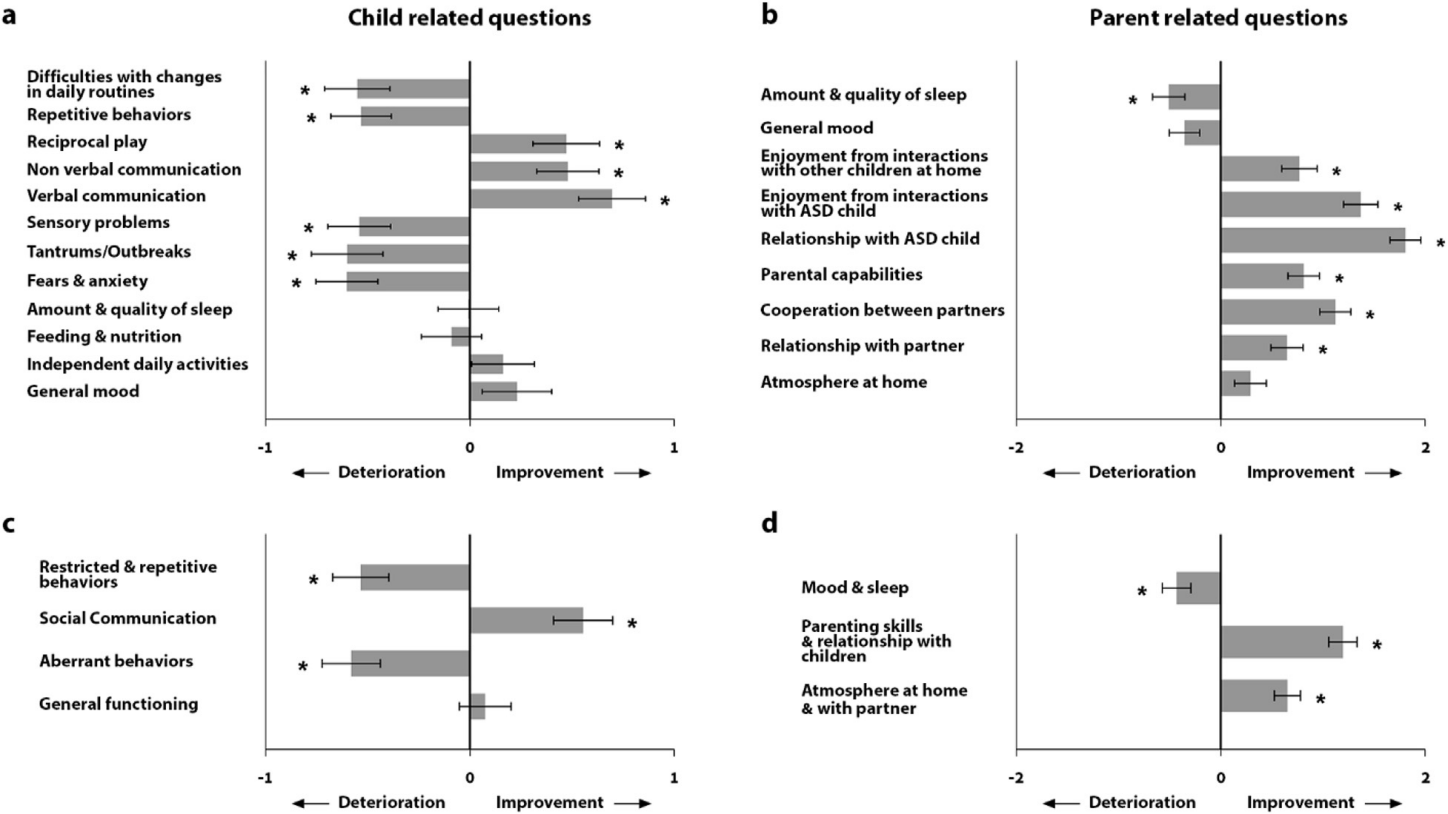
Parent perceived impact of six-week home-quarantine period. Mean ratings on 12 child-related questions (a) and 9 parent-related questions (b). Positive values indicate parental report of improvement and negative values indicate deterioration. Items were sub-grouped into four child-related domains (c) and three parent-related domains (d). Error bars: standard error of the mean across participants. Asterisks: significant impact (two-tailed t-test, *p* < 0.05, Bonferroni corrected).

Parent retrospective reports indicated that home-quarantine had significant impact on their children with ASD (Figure 1A). This included significant deterioration in the child's sensory problems (*t*(264) = -3.5, *p* < 0.001), fears and anxiety (*t*(264) = -3.9, *p* < 0.001), the amount and severity of tantrums/outbreaks (*t*(263) = -3.4, *p* < 0.001), the child's ability to cope with changes in daily routines (*t*(263) = -3.5, *p* < 0.001), and the amount and severity of repetitive/stereotypical behaviors (*t*(261) = -3.6, *p* < 0.001). In contrast, parents reported significant improvements in their child's reciprocal play (*t*(263) = 2.9, *p* = 0.004), non-verbal communication (*t*(265) = 3.1, *p* = 0.002), and verbal communication (*t*(263) = 4.2, *p* < 0.001). There were no significant changes in the child's general mood (*t*(265) = 1.3, *p* = 0.18), feeding (*t*(266) = -0.6, *p* = 0.54), quantity and quality of sleep (*t*(266) = -0.05, *p* = 0.96), or independence in daily activities (*t*(266) = 1.1, *p* = 0.29).

Analysis of the 9 parent-related questions also revealed important dissociations across items (Figure 1B). Parents perceived significant negative impact on the amount and quality of their sleep (*t*(265) = -3.2, *p* = 0.002) and a trend of negative impact on their general mood (*t*(265) = -2.3, *p* = 0.02, not significant after Bonferroni correction). However, they reported significant improvements in the quality of the relationship with their child with ASD (*t*(265) = 11.9, *p* < 0.001) as well as more enjoyment from time spent with their child with ASD (*t*(266) = 8.1, *p* < 0.001) and with their other children (*t*(261) = 4.4, *p* < 0.001). Parents also reported improvements in their parenting skills (*t*(266) = 5.3, *p* < 0.001), parental cooperation (*t*(256) = 7.4, *p* < 0.001), and the relationship with their domestic partner (*t*(250) = 4.1, *p* < 0.001). A trend of positive impact was also apparent in the general atmosphere at home (*t*(265) = 1.9, *p* = 0.06). Note that the largest impact of the quarantine across all reported items was the positive change in the parent's relationship with their child with ASD (Figure 1B).

We then grouped conceptually and theoretically related questions with high internal consistency (see Methods) into 4 child-related domains (Figure 1C) and 3 parent-related domains (Figure 1D) and re-ran our analyses after averaging the ratings in each domain. Significant parent perceived deterioration was apparent in the child-related RRB domain (*t*(266) = -3.9, *p* < 0.001) and aberrant behaviors domain (*t*(264) = -4.1, *p* < 0.001). In contrast, significant improvement was apparent in the social communication domain (*t*(266) = 3.8, *p* < 0.001). No significant change was apparent in the general functioning domain (*t*(266) = 0.59, *p* = 0.56). Significant deterioration was apparent in the parent-related mood and sleep domain (*t*(265) = -3.1, *p* = 0.002), while significant improvements were apparent in the parenting skills and relationship with children domain (*t*(266) = 8.6, *p* < 0.001) as well as the atmosphere at home domain (*t*(267) = 5.1, *p* < 0.001).

### 3.1 Children's age and gender

**Figure 2:**
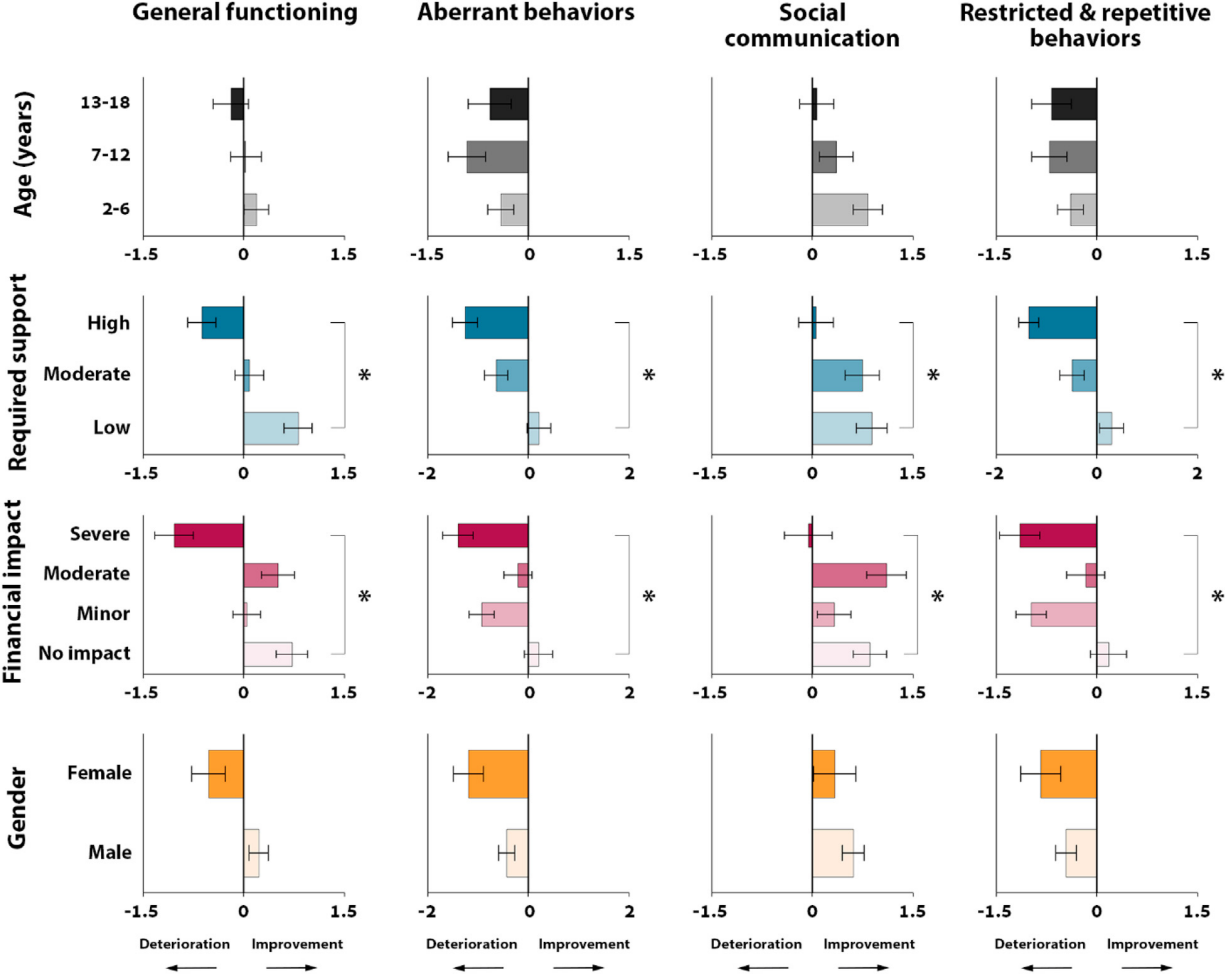
Perceived quarantine impact on children with ASD, stratified into subgroups according to the child's age, level of required support, economic hardships, and gender. Columns present mean ratings in each child-related domain. **1**^**st**^**row:** Stratification by children's age into pre-school (2-6 years-old), school-age (7-12 years-old) and adolescent (13-18 years-old) groups. **2**^**nd**^**row:** Stratification by the level of support that the child with ASD usually requires (high, moderate, and low). **3**^**rd**^**row:** Stratification by economic hardships into severe (>40% decrease), moderate (20–40% decrease), minor (<20% decrease), and no impact (no decrease) groups. **4**^**th**^**row:** Stratification by sex of the child. **Error bars:** standard error of the mean across participants. **Asterisks:** significant differences in one-way ANOVA analysis (*p* < 0.05).

**Figure 3:**
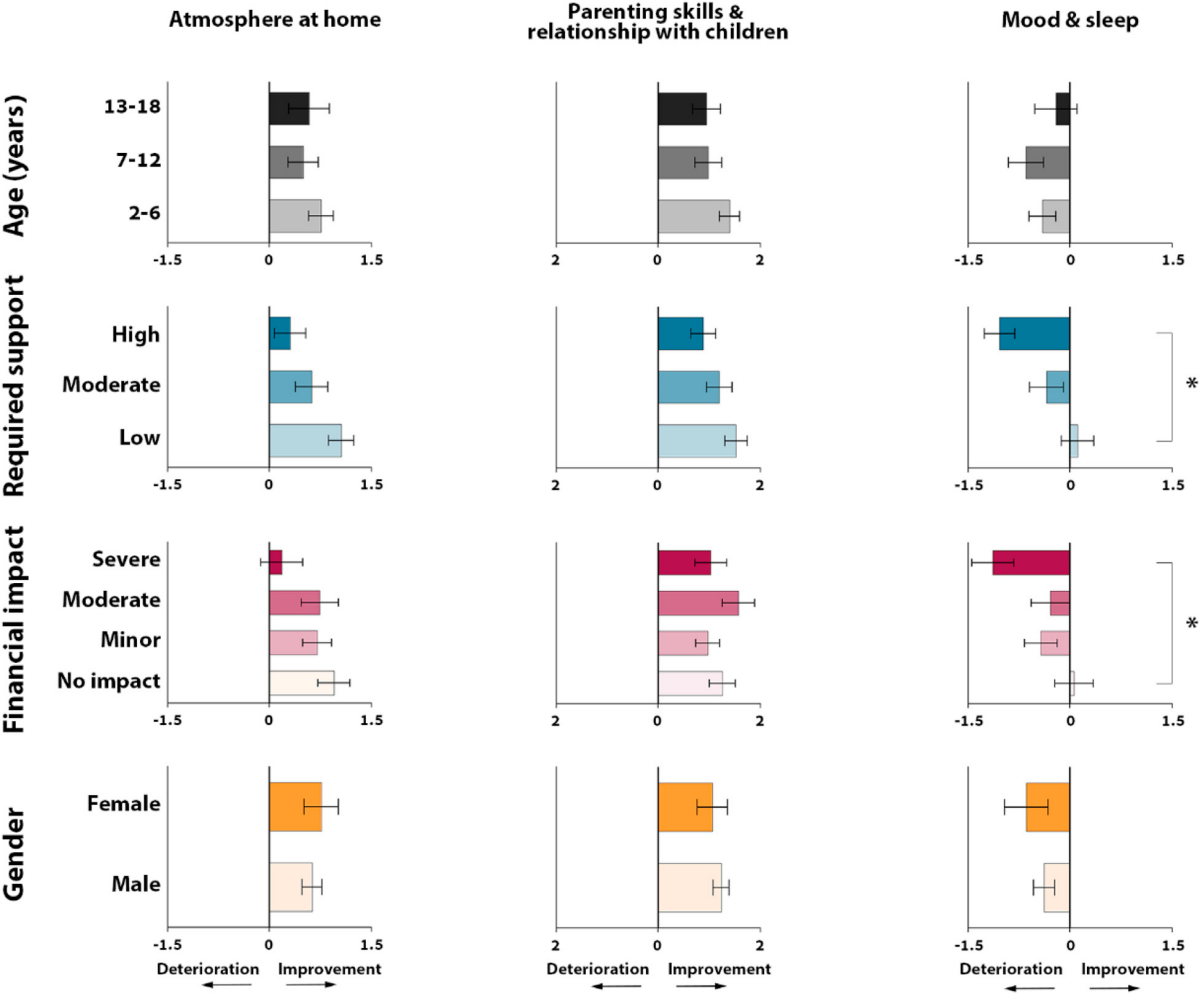
Perceived quarantine impact on parents when stratifying subgroups according to the child's age, level of required support, financial impact, and gender. Columns present mean ratings of each parent-related domain. **1**^**st**^**row:** Stratification by the child's age into pre-school (2-6 years-old), school-age (7-12 years-old) and adolescent (13-18 years-old) groups. **2**^**nd**^**row:** Stratification according to the level of support that the child with ASD usually requires (high, moderate, and low). **3**^**rd**^**row:** Stratification according to financial impact into severe (>40% decrease), moderate (20–40% decrease), minor (<20% decrease), and no impact (no decrease) groups. **4**^**th**^**row:** Stratification by sex of the child. **Error bars:** standard error of the mean across participants. **Asterisks:** significant differences in one-way ANOVA analysis (*p* < 0.05).

To examine the relationship between perceived impact and the children's age, we compared parent ratings of 2–6-year-old children (pre-school), 7–12-year-old children (school age), and 13-18 year-old children (adolescents). A one-way ANOVA analysis was performed to determine significant differences across age groups (i.e., independent variable). This analysis did not reveal any significant differences in the ratings of the three age groups in either child (*F*(2,263 < 2.3, *p* > 0.1, Figure 2) or parent (*F*(2,264) <1.2, *p* > 0.3, Figure 3) related domains. Likewise, there were no significant differences across parental reports of boys and girls with ASD in any of the child (Figure 2) or parent (Figure 3) related domains (*p* > 0.1; two-sample t-tests).

### 3.2 Children's level of required support

Approximately one third of parents reported that their child with ASD required high, moderate, or low levels of support. A one-way ANOVA analysis was performed to determine significant differences across different levels of required support (i.e., independent variable). This analysis revealed significant differences across parent ratings of children with different levels of required support (Figure 2). Significant differences were apparent in ratings of general functioning (*F*(2,263) = 11.5, *p* < 0.001), aberrant behaviors (*F*(2,261) = 9.3, *p* < 0.001), social communication (*F*(2, 263) = 3.2, *p* = 0.04), and RRB (*F*(2,260) = 12.9, *p* < 0.001) symptoms. Post-hoc analyses revealed that children with high support requirements exhibited significantly lower ratings than children with low support requirements in all four domains (*p* < 0.05; post-hoc Tukey's test).

Similar results were also apparent in the parent-related domains (Figure 3). One-way ANOVA analyses revealed significant differences across groups in the parental mood and sleep domain (*F*(2,262) = 5.8, *p* = 0.003). A trend in the same direction was also apparent in the atmosphere at home (*F*(2,262) = 2.9, *p* = 0.06). Post-hoc analyses revealed that parents of children with high support requirements reported significantly larger deterioration in the mood and sleep domain than parents of children with low support requirements (*p* = 0.002, Tukey's test). These parents also reported significantly less improvement in the parenting skills and relationships with children domain (*p* = 0.04, Tukey's test).

### 3.3 Financial impact of home-quarantine

The degree of financial impact experienced by the families also had a significant effect on parental ratings. Approximately 23% of the families reported a severe reduction in income (>40%), 23% reported a moderate reduction in income (20–40%), 29% reported a minor reduction in income (<20%), and 25% reported no reduction in income. A one-way ANOVA analyses was performed to compare ratings across families with different income reductions (i.e., independent variable). This analysis revealed significant differences across groups in the ratings of all child-related domains (Figure 2): general functioning (*F*(3,262) = 9.9, *p* < 0.001), aberrant behaviors (*F*(3,260) = 6.5, *p* < 0.001), social communication (*F*(3,262) = 3.2, *p* = 0.02), and RRB (*F*(3,259) = 5.6, *p* < 0.001) symptoms. Post-hoc Tukey's tests revealed that children in families with severe financial impact exhibited significantly lower ratings than children in families with no financial impact in general functioning (*p* < 0.001), aberrant behaviors (*p* < 0.001), and RRB symptoms (*p* = 0.004). A similar weak trend was also apparent in the social communication symptoms domain (p = 0.12). These findings suggest that severe reductions in family income had negative impact on children's ratings in all domains.

Similar findings were apparent only in one of the parent-related domains (Figure 3): parental mood and sleep (*F*(3,261) = 3.1, *p* = 0.03). Parents in families with severe financial impact had significantly lower mood and sleep ratings than parents in families with no financial impact (*p* = 0.015; post-hoc Tukey's test). One-way ANOVA analyses did not reveal significant differences in the other parent-related domains (*F*(3,263)<1.5, *p* > 0.2). Note that parents consistently reported a significant improvement in parenting skills and relationship with children, regardless of financial impact (*p* < 0.002, one-sample t-test).

### 3.4 Amount and efficiency of online consultations

**Figure 4:**
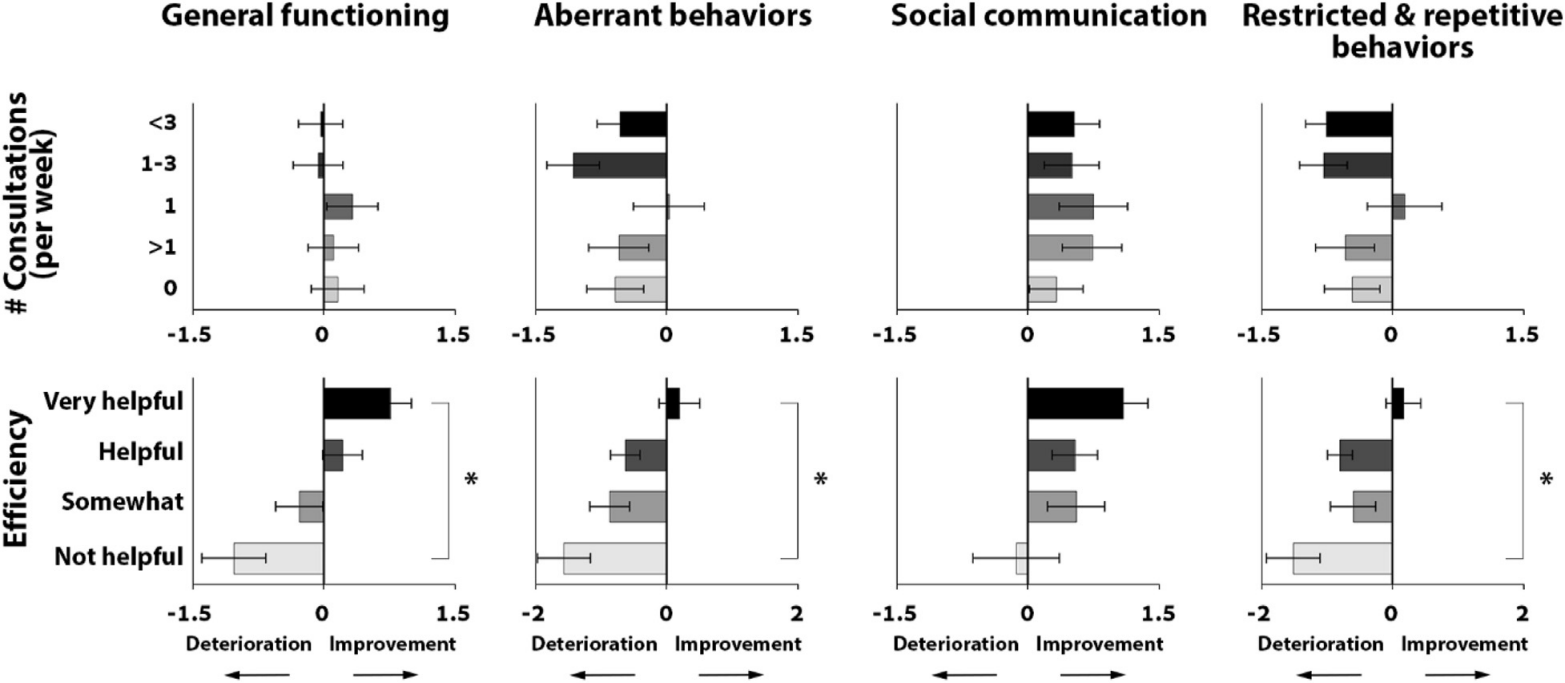
Perceived impact of online consultations on children with ASD. **Top row:** Stratification according to the number/quantity of online consultations that the parents or child received per week (ranging from 0 to >3 times per week). **2**^**nd**^**row:** Stratification according to the perceived efficacy of online consultations as reported by the parents (ranging from not helpful to very helpful). **Error bars:** standard error of the mean across participants. **Asterisks:** significant differences in one-way ANOVA analysis (*p* < 0.05).

Parents also reported the number of online consultations per week that they and their children received during the quarantine from the children's educational teams and therapists (Figure 4). Approximately 80% of the families reported receiving online consultations with 22% of the families receiving <1 consultation per week, 18% receiving 1 consultation per week, 27% receiving 1–3 consultations per week, and 33% receiving >3 consultations per week. A one-way ANOVA analysis was performed to identify differences across groups receiving different numbers of consultations (i.e., independent variable). This analysis did not reveal significant differences in any of the child-related domains across groups (*F*(4, 258) <1.3, *p* > 0.26). Hence, the quantity of online services did not affect parental ratings in any of the examined domains.

Families that received online consultations also rated their effectiveness. One-way ANOVA analyses revealed that parents who thought that online consultations were more effective, reported that their children improved more (Figure 4) in their general functioning (*F*(3,210) = 7.2, *p* < 0.001), aberrant behaviors (*F*(3,209) = 5.2, *p* = 0.002) and RRB symptoms (*F*(3,208) = 5, *p* = 0.002). A similar trend was also apparent in the social communications domain (*F*(3,208) = 2.1, *p* = 0.1). Post-hoc Tukey's tests revealed that parents who felt that online consultations were very helpful reported that their children improved significantly more than parents who reported that online services were not helpful. This was apparent in ratings of general functioning (*p* < 0.001), aberrant behaviors (*p* < 0.001), and RRB symptoms (*p* < 0.001).

In total, 47% of all participating parents reported that online consultations were helpful or very helpful, demonstrating the success of these services during the home-quarantine period.

## 4 Discussion & implications

Our results demonstrate that parent perceived the initial Covid-19 home-quarantine period as having both negative and positive impact on their wellbeing and that of their children with ASD. While parents reported that their general mood and sleep deteriorated during the quarantine, they also reported that their family relationships with spouse and children as well as their parenting skills improved (Figure 1). Furthermore, while parents reported that their children with ASD exhibited increased tantrums, anxiety, and elevated RRB symptoms, they also reported improvements in the children's social and communication abilities (Figure 1). Hence, despite the many difficulties and challenges of home-quarantine, many parents perceived improvements in family relationships and social interactions during this period, particularly in families of children with ASD who required lower levels of support and families that were not affected financially (Figures 2 and 3).

Available online support services were reported helpful by almost half of the families, but there was no relationship between the amount of services and their perceived impact (Figure 4). Further development of more structured online support services that enhance the positive impact of home-quarantine, perhaps by further empowering parents, are highly warranted. This may be particularly important for families of children who require more support and those experiencing financial hardships.

### 4.1 Negative impact of home-quarantine

Parent reports regarding the negative psychological effects of home-quarantine on children with ASD were expected [15, 16, 17]. Recent studies have indeed reported that Covid-19 lockdowns have caused an increase in the severity of aberrant behaviors [25, 36], sleep difficulties, and lethargy [26] in children with ASD as well as increased levels of stress and anxiety in both children with ASD and their parents [23, 24]. Furthermore, parents of children with ASD experienced a larger decrease in quality of life compared to parents of typically developing children [37]. Our results are in agreement with these reports and demonstrate deterioration in the general mood and sleep quality of the parents as well as an increase in the severity of aberrant behaviors and RRB symptoms of their children with ASD (Figure 1).

Increased RRB symptoms during home quarantine may reflect an adaptive coping mechanism whereby children with ASD engaged in more repetitive behaviors to reduce their anxiety [38, 39]. Alternatively, the reported change may be due to increased parent sensitivity given the extensive continuous time that parents spent with their children at home. Another alternative is that increased RRB symptoms were due to the sudden cessation of external intervention services, particularly for children in families with higher levels of accommodation [40].

### 4.2 Positive impact of home-quarantine

Despite the challenges described above, parents of children with ASD reported that home-quarantine also had positive impact in several domains. First, parents reported that the social communication abilities of their child with ASD improved, including verbal and non-verbal communication, and reciprocal play. This is in agreement with recent reports of improvement in communication [27], language, and cognitive abilities [41] as well as adaptive behaviors [28] of children with ASD following Covid-19 lockdowns.

We speculate that home-quarantine periods may have enabled parents and children to get to know each other better and improve their communication. In addition, parents may have been more available to participate in educational, social, and play activities, which are particularly beneficial for children's emotional, social, and cognitive development [18, 19, 20, 21]. Indeed, parents also reported improvement in their parenting abilities (Figure 1) [27]. Nevertheless, it is not trivial that such positive changes were reported by parents in the context of home-quarantine during a global pandemic and despite their reports of deterioration in RRB symptoms and aberrant behaviors.

A second positive impact of home-quarantine was apparent in family relationships and cooperation between domestic partners (Figure 1). In the context of relationship science, Covid-19 has been described as an external stressor that is likely to have negative impact on the stability and quality of couples' relationships [42]. However, our findings suggest that most parents of children with ASD, despite their preexisting vulnerabilities [43], exhibited surprising resilience during the home-quarantine period. Note that these positive changes in couple's relationships were reported despite negative changes in their general mood and sleep, perhaps reflecting a response of family solidarity in the face of an external threat [44].

### 4.3 Variables affecting parent perceptions of quarantine impact

While the age and gender of children with ASD did not have a significant effect on parent ratings in any of the examined domains, the level of the child's required support and the magnitude of economic hardships did affect parent ratings. Ratings of children with ASD who required high levels of support were consistently lower than ratings of children with ASD who required low levels of support in all child-related domains (Figure 2). This suggests that quarantine related hardships were larger in families where children with ASD had more severe symptoms. Nevertheless, parents of all children with ASD still reported improvement in their parenting skills and the relationship with their child with ASD as well as modest improvement in the atmosphere at home and relationship with partner (Figure 3). This further highlights the resilience of many parents of children with ASD who reported improvement in their family relationships despite deterioration in their mood and sleep.

Similarly, parent ratings in families that experienced severe financial impact (i.e., >40% drop in income) were consistently lower than those of parents in families that were not affected financially, across all child-related domains. This suggests that parents perceived the impact of quarantine as being generally more negative when accompanied by financial hardships, which may act as a strong additional source of stress and anxiety [12, 13]. However, most parents still reported improvement in their parenting skills and the relationship with their child with ASD, regardless of the magnitude of economic hardships (Figure 3).

### 4.4 Online services

During the home-quarantine period many daycare, pre-school, and school settings shifted quickly to provide improvised online services to families of children with ASD. This included online video consultations with the child and parents as well as other interactions through social media and other communication channels. There was considerable variability in the number of online consultations that different families received. Interestingly, child ratings did not differ across families who received different amounts of online consultations (Figure 4), suggesting that the frequency of consultations were not associated with their effectiveness. Nevertheless, almost half of the parents reported that online consultations were either helpful or very helpful and these parents also reported larger improvements in their children's behaviors and abilities. In agreement with recent studies [45], improvised online services seem to have successfully helped approximately half of the families. We speculate that developing structured online services that empower parents by enhancing parenting skills may potentially increase their efficacy and increase the positive impact of home-quarantine periods caused by pandemics, natural disasters, or other reasons.

### 4.5 International perspective

Different populations around the world may have perceived the impact of Covid-19 in different ways. Specifically, the population of Israel has considerable experience in coping with security threats that escalate every several years into situations that may resemble Covid-19 lockdowns. For example, during periods of missile attacks, large sections of the Israeli population stay at home and remain close to bomb-shelters. Hence, parent ratings of home-quarantine impact in Israel may differ from those of other populations. Indeed, studies comparing the Israeli general population with populations in the US, Poland, and Canada have reported that Israeli participants reported less anxiety and depression following the Covid-19 outbreak [46, 47].

### 4.6 Limitations

The current study had several limitations. First, due to privacy constraints, the online questionnaire was filled out anonymously by parents. Hence, we have no way of verifying the accuracy of the supplied information and do not have verified clinical data regarding diagnosis or measures of symptom severity from participating children. Second, the questionnaire relied on parental judgments of retrospective change over the six-week home-quarantine period, which may suffer from optimism-bias [48] and other parent report biases [49, 50]. Third, findings from this study are relevant to the initial home-quarantine period at the beginning of the Covid-19 outbreak. We speculate that parent ratings may change over time as the pandemic continues and external stressors become chronic. Fourth, the questionnaire used in the current study was not standardized or validated on a large population and its psychometric properties are, therefore, unknown.

### 4.7 Conclusions

The current study adds to accumulating evidence demonstrating that parents perceived home quarantine periods as having both positive and negative impact on their well-being and that of their children with ASD. Despite the clear difficulties and challenges created by Covid-19, the results suggest that isolating families at home may also offer a unique opportunity for improving family relationships as well as social and communication abilities. Acknowledging the positive aspects of home quarantine may help in designing more effective online support services that empower parents during such periods. Such services seem particularly important for families of children with ASD who require more support and those experiencing financial difficulties, where home quarantine is perceived to have a larger negative impact.

## Declaration

### Author contribution statement

Ayelet Arazi: Conceived and designed the experiments; Performed the experiments; Analyzed and interpreted the data; Wrote the paper. Judah Koller; Ditza A. Zachor; Ofer Golan; Yair Sadaka; Dganit Eytan; Orit Stolar; Naama Atzaba-Poria; Hava Golan; Idan Menashe; Gal Meiri; Lidia V. Gabis:Conceived and designed the experiments; Wrote the paper.

Ilan Dinstein: Conceived and designed the experiments; Analyzed and interpreted the data; Wrote the paper.

### Funding statement

This study was supported by 10.13039/501100003977Israel Science Foundation [1150/20].

Prof. Ilan Dinstein was supported by Ministry of Science and Technology, Israel & Azrieli Foundation [Autism Knowledge Center & National Autism Center].

### Data availability statement

Data will be made available on request.

### Declaration of interest's statement

The authors declare no conflict of interest.

### Additional information

No additional information is available for this paper.

## Appendix A Supplementary data

Covid 19 impact on Autism Supp Info Heliyon Rev.docx Covid 19 impact on Autism Supp Info Heliyon Rev.docx
