# Supplementary material for: Home-quarantine during the initial Covid-19 outbreak in Israel: parent perceived impact on children with ASD
Source: Heliyon. 2022 Jun 8;8(6):e09681. doi: 10.1016/j.heliyon.2022.e09681 (PMC9176182; doi:10.1016/j.heliyon.2022.e09681)
Supplement: Covid 19 impact on Autism Supp Info Heliyon Rev.docx [file mmc1.docx]

**Home-quarantine during the initial Covid-19 outbreak in Israel: Parent perceived impact on children with ASD – Supplementary Materials**

Ayelet Arazi^1,2^, Judah Koller^3^, Ditza A. Zachor^4,5^, Ofer Golan^6,7,8^, Yair Sadaka^9^, Dganit Eytan^10,11^, Orit Stolar^5^, Naama Atzva-Poria^12^, Hava Golan^13,2^, Idan Menashe^14,2^, Gal Meiri^15,2^, Lidia V. Gabis^4,16,17^, Ilan Dinstein^12,2,1^

1. Department of Cognitive & Brain Sciences, Ben Gurion University of the Negev, Beer Sheva, Israel
2. Azrieli National Centre for Autism and Neurodevelopment Research, Ben Gurion University of the Negev, Beer Sheva, Israel
3. Seymour Fox School of Education, The Hebrew University of Jerusalem, Jerusalem, Israel
4. Sackler Faculty of Medicine, Tel Aviv University, Tel Aviv, Israel
5. The Autism Center/ALUT, Shamir (Assaf Harofeh) Medical Center, Israel
6. Autism Treatment and Research Center – Association for Children at Risk
7. Department of Psychology, Bar-Ilan University, Ramat Gan, Israel
8. Autism Research Centre, Department of Psychiatry, University of Cambridge, UK
9. Neuro-Developmental Research Centre, Beer Sheva Mental Health Centre, Ministry of Health, Beer Sheva, Israel
10. ALUT – The Israeli Society for Children and Adults with Autism, Ramat Gan, Israel
11. School of Education, Bar-Ilan University, Ramat Gan, Israel
12. Department of Psychology, Ben Gurion University of the Negev, Beer Sheva, Israel
13. Department of Physiology and Cell Biology, Faculty of Health Sciences, Ben Gurion University of the Negev, Beer Sheva, Israel
14. Department of Public Health, Ben Gurion University of the Negev, Beer Sheva, Israel
15. Pre-School Psychiatry Unit, Soroka University Medical Center, Beer Sheva, Israel
16. Sagol School of Neuroscience, Tel Aviv University, Tel Aviv, Israel
17. Child Development Center, Edmond and Lily Safra Children’s Hospital, Sheba Medical Center, Tel Hashomer, Israel

Questionnaire:

**Part 1 - General information:**

| I am:   - Mother - Father | The gender of my child with ASD is:   - Male - Female |
| --- | --- |
| Age of my child with ASD: ______ | The age of the parent filling the questionnaire: ______ |
| The level of daily support my child requires:   - Low - Medium - High | Before the quarantine began my child went to:   - School - Kindergarten - Daycare - Stayed at home - Other |
| My child with ASD normally attends the following education setting:   - Special education - Communication class - Regular class with an assistant - Regular class without an assistant - Other | Before the quarantine my child received the following services (please mark all relevant options):   - Speech or language therapy. Hours per week: ______ - Occupational therapy. Hours per week: ______ - Physical therapy. Hours per week: ______ - Psychological therapy. Hours per week: ______ - Music/art/other therapy. Hours per week: ______ |

**Part 2 – Impact of the quarantine on the parents:**

From the beginning of quarantine, at least one parent was permanently at home:

- Yes
- No

The financial impact of the quarantine on our family’s income was:

- Severe (decrease of more than 40%)
- Moderate (decrease of 20-40%)
- Minor (decrease of less than 20%)
- No impact

Please rate the impact of the quarantine period in the following domains on a scale of -5 (very negative impact) to 5 (very positive impact)

very negative impact no impact very positive impact

|  | -5 | -4 | -3 | -2 | -1 | 0 | 1 | 2 | 3 | 4 | 5 |
| --- | --- | --- | --- | --- | --- | --- | --- | --- | --- | --- | --- |
| Atmosphere at home |  |  |  |  |  |  |  |  |  |  |  |
| Relationship with domestic partner (if relevant) |  |  |  |  |  |  |  |  |  |  |  |
| Cooperation between parents in caring for the child with ASD |  |  |  |  |  |  |  |  |  |  |  |
| Parental capabilities (i.e., feeling failure or success as a parent) |  |  |  |  |  |  |  |  |  |  |  |
| Relationship with child with ASD |  |  |  |  |  |  |  |  |  |  |  |
| Enjoyment from interactions with child with ASD |  |  |  |  |  |  |  |  |  |  |  |
| Enjoyment from interactions with other children at home (if relevant) |  |  |  |  |  |  |  |  |  |  |  |
| General mood |  |  |  |  |  |  |  |  |  |  |  |
| Amount and quality of sleep |  |  |  |  |  |  |  |  |  |  |  |

**Part 3 – Impact of the quarantine on the child with ASD**

Please rate the impact of the quarantine on your child with ASD in the following domains using a scale of -5 (very negative impact) to 5 (very positive impact):

very negative impact no impact very positive impact

|  | -5 | -4 | -3 | -2 | -1 | 0 | 1 | 2 | 3 | 4 | 5 |
| --- | --- | --- | --- | --- | --- | --- | --- | --- | --- | --- | --- |
| General mood (emotional state of the child) |  |  |  |  |  |  |  |  |  |  |  |
| Performance of daily activities (e.g., independently brushing teeth or using the toilet) |  |  |  |  |  |  |  |  |  |  |  |
| Eating and nutrition |  |  |  |  |  |  |  |  |  |  |  |
| Amount and quality of sleep |  |  |  |  |  |  |  |  |  |  |  |
| Fears and anxiety |  |  |  |  |  |  |  |  |  |  |  |
| Amount and severity of outbreaks/tantrums |  |  |  |  |  |  |  |  |  |  |  |
| Sensory problems (e.g., sensitivity to noise or touch) |  |  |  |  |  |  |  |  |  |  |  |
| Verbal communication |  |  |  |  |  |  |  |  |  |  |  |
| Nonverbal communication (e.g., eye contact and facial or body gestures) |  |  |  |  |  |  |  |  |  |  |  |
| Reciprocal play |  |  |  |  |  |  |  |  |  |  |  |
| Amount and severity of stereotypical/repetitive behaviors (e.g., hand flapping) |  |  |  |  |  |  |  |  |  |  |  |
| Difficulties with changes in daily routine |  |  |  |  |  |  |  |  |  |  |  |

**Part 4 – Use of online tools for receiving services**

How often during quarantine did you receive guidance and consultations about autism related issues (including direct intervention meetings with your child) by video chats or phone calls from professionals in your child’s educational setting?

- More than 3 times a week
- 1-3 times a week
- Once a week
- Rarely
- Not at all

In which domains did you receive such guidance/consultations?

- Speech and language therapy
- Occupational therapy
- Psychology
- Music/art/animal/hydro/other therapy
- Other ______

To what degree were these online consultations effective?

- Very helpful
- Helpful
- Somewhat helpful
- Not helpful at all

How often did you receive autism related information via social media (e.g., WhatsApp, Facebook)?

- Dozens of times a day
- Several times a day
- Once a day
- Several times a week
- Rarely
- Never

To what degree was the information from social media helpful in dealing with quarantine difficulties?

Made it more difficult was very helpful

| 0 | 1 | 2 | 3 | 4 | 5 | 6 | 7 | 8 | 9 | 10 |
| --- | --- | --- | --- | --- | --- | --- | --- | --- | --- | --- |

Have you actively searched online for information and guidance regarding the challenges of caring a child with autism during quarantine?

- Yes
- No

If yes, have you found useful information in Hebrew?

- Yes. Please specify the name of the website: ______
- Partially
- No

Is there any other type of online support which could have helped you deal with autism challenges during the quarantine? ____________________________________________________
